# Spatial Modelling of Risk Factors for Malaria Prevalence in SNNP Regional State, Ethiopia

**DOI:** 10.4314/ejhs.v31i4.7

**Published:** 2021-07

**Authors:** Shammena Aklilu Toma, Baleh Wubejig Eneyew, Goshu Ayele Taye

**Affiliations:** 1 Department of Statistics, College of Natural and Computational Sciences, Hawassa University, Hawassa, Ethiopia; 2 Department of Statistics, College of Natural and Computational Sciences, Kotebe Metropolitan University, Addis Ababa, Ethiopia

**Keywords:** Spatial Dependency, Spatial Lag Model, Spatial Error Model, Malaria Prevalence, Pathologic character

## Abstract

**Background:**

Malaria is one of the most severe public health problems worldwide with 300 to 500 million cases and about one million deaths reported to date of which 90% were from world health organization (WHO) Sub Saharan Africa (SSA) countries. The purpose of this study was to explore the spatial distribution of malaria parasite prevalence (MPP) among districts of Southern Nations Nationalities and Peoples Regional State (SNNRS) in Ethiopia by using 2011 malaria indicator survey (MIS) data collected for 76 districts and to model its relationship with different covariates.

**Method:**

Exploratory spatial data analysis (ESDA) was conducted followed by implementation of spatial lag model (SLM) and spatial error model (SEM) in GeoDa software. Queen contiguity second order type of spatial weight matrix was applied in order to formalize spatial interaction among districts.

**Results:**

From ESDA, we found positive spatial autocorrelation in malaria prevalence rate. Hot spot areas for MPP were found in the eastern and southeast parts of the region. Relying on specification diagnostics and measures of fit, SLM was found to be the best model for explaining the geographical variation of MPP. SLM analysis demonstrated that proportion of households living in earth/local dung plastered floor house, proportion of households living under thatched roof house, average number of rooms/person in a given district, proportion of households who used anti-malaria spray in the last 12 months before the survey, percentage household using mosquito nets and average number of mosquito nets/person in a given district have positive and statistically significant effect on spatial distribution of MPP across districts of SNNPRS. Percentage of households living without access to radio and television has negative and statistically significant effect on spatial distribution of MPP across districts of MPP.

**Conclusion:**

Malaria is spatially clustered in space. The implication of the spatial clustering is that, in cases where the decisions on how to allocate funds for interventions needs to have spatial dimension.

## Introduction

Malaria is a deadliest parasite disease for human being which is caused by Plasmodium parasite infection. Malaria is one of the most severe public health problems worldwide with 300 to 500 million cases and about one million deaths reported to date of which 90% were from world health organization (WHO) Sub Saharan Africa (SSA) countries (1).

In developing countries, malaria is one of the major tropical disease (usually called disease of poor people) adversly affecting its economic development and is the fourth leading cause of death for children under the age of five years (2).

Malaria is the major concern in Ethiopia since it is one of the leading causes of morbidity and mortality. Despite the current efforts to control malaria in Ethiopia, the situation has not improved mainly due to the incresing problems of parasite resistance to relatively cheaper anti-malarial drugs, vector resistance to insecticide, low coverage of malaria preventive services, poor access to health care, rudilmentary health service infrastructure, large population movement and limited financial and human resource (3,4).

Eventhough the exact number of people getting sick and dying of malaria every year in Ethiopia is unknwon, it is known that tens of thousands of people die due to malaria every year and that morbidity and mortality rates increse dramatically during the epidemics. The distribution of malaria in Ethiopia is not uniform. There are areas where the risk of malaria is high and there are areas where the risk of malaria is low despite 75% of the the country is malarious with about 68% of the total population living in areasat risk of malaria (5,6).

Due to unstable and seasonal patter of malaria transmission, the protective immunity of the population is generally low and all age groups are at risk of infection and disease. Some small-scale studies have docummented on malaria parasite prevalence betwwen 10.4%–13.5% in Gambela, 7.6%–14.1% in Tigray, 4.6% in Amhara, 0.9% in Oromia and 5.4% in Southern Nations, Nationalities and Peoples Regional State (SNNPRS) in all age groups. In addition to its impcat on health, malaria imposes a heavy economic barden on individuals and reduces economic output (7).

Different studies use different categories of covariates in modeling malaria trasmission intensity, along with its temporal and spatial distribution in SSA. Climatic and environmental, socio-demographic, and malaria intervention covariates were usually used. The most common covariates in the environmental domain were temperature, humidity and rainfall (8, 9, 10, 11) while socio-demographic covariates include socioeconomic status (12), gender (14), age (14), population size (14, 15), livestock ownership (16), wealth index (12, 16) and building/housing material (14, 16).

In addition, common covariates under malaria intervention domain include insecticide-treated bed nets (ITN) ownership (13, 18), indoor residual spraying (IRS) (12, 13), artemisinin-based combined therapy (ACTs) (12, 17), treatment seeking rate (17, 18) and transmission seasonality (13, 17, 18).

The levels of malaria risk and transmission intensity exhibit significant spatial and temporal variability related to variations in climate, altitude, topography, and human settlement pattern. The spatial and temporal patterns of malaria transmission at the local level (fixed spatial scale) in semi-arid and highland regions in Africa, and particularly in Ethiopia, have not been well investigated or accurately defined. Such research is needed in developing dynamic and area-specific risk maps to identify locations and populations at highest risk for appropriate planning and implementation of targeted and epidemiologically sound preventive and control measures

The existing malaria risk maps have limited operational use to support programmatic activities since they were produced at coarse spatial scales (at continental and country levels). They are largely based on expert opinion, on climate-based models, and specific geo-referenced point prevalence data. In this context, geographical information systems (GIS), remote sensing satellite imagery, geospatial techniques, and spatial statistics provide new methodologies and solutions to analyze the epidemiological and ecological context of malaria and other infectious diseases (19, 20).

Having this background, this study is intended to assess spatial dependence of malaria distribution and to fit suitable spatial regression model by incorporating environmental, socio-demographic and malaria intervention covariates across districts of SNNPRS in Ethiopia.

## Methods

**Description of the Study Area and Population**: SNNPRS is located in the southern and south-western part of Ethiopia. It is bordered with Kenya in south, Sudan in southwest, Gambella region in northwest and surrounded by Oromia region in northwest, north and east directions. It is one of the largest regions in Ethiopia, accounting for more than 10% of the country's land area. Based on 2007 census conducted by the Central Statistical Agency (CSA) of Ethiopia, the region has an estimated total population of 14,929,548 of whom 13,433,991(89.98%) are rural inhabitants while 1,495,557(10.02%) are urban which makes the region overwhelmingly rural. The region is administratively divided into 13 administrative zones, 133 Woredas and 3512 Kebeles (the smallest administrative units in Ethiopia), and its capital is Hawassa city (21).

**Data collection procedure**: This work was based on data available from the 2011 Ethiopian malaria indicator survey (EMIS-2011) of Ethiopian Public Health Institution (EPHI) which was conducted from October 2011 GC to December 2011 GC. EMIS-2011 is a large, nationally representative survey of coverage of key malaria control interventions, treatment-seeking behavior and malaria prevalence (22).

A stratified two-stage cluster sample design was implemented in order to identify sample households. Census enumeration areas (EAs) were the primary sampling units (PSUs). Households within selected EAs were second-stage sampling units. Spatially aggregated data across SNNPRS on all variables were extracted and used for analysis Shape file map was obtained from Finance and Economic Development office of SNNPRS.

**Variables considered in the study**: The dependent variable is malaria parasite prevalence (MPP) per districts of SNNPRS which indicates the proportion of positive malaria diagnosis test (RDT) result per districts of SNNPRS. Moreover, the following covariates were analyzed in the study:

ALTITUDE: mean altitude of a district above sea level in meter

DRW_100: proportion of households having piped water

TOICOV_100: proportion of households having protected toilet

ELEC_100: proportion of households having access to electricity

RDTV_100: percentage of households having no access to radio and television

NRM: average number of rooms/person in a given district

WAL_100: proportion of households whose living house wall is mud/stick/wood

ROF_100: proportion of households living under thatched roof house

FLR_100: proportion of households living in earth/local dung plastered floor house

UMN_100: percentage of households using mosquito nets

NMN: average number of mosquito nets/person in a given district

ANMSP_100: proportion of households who used anti-malaria spray in the last 12 months before the start of EMIS-2011

NHHDM: average number of household members (average family size)

WEALTHIX: average wealth quintile of households in a given district

**Data analysis**

**Geospatial analysis**: Geospatial data was analyzed by geographic locations to evaluate geospatial distribution and identify hotspot areas for MPP at district level. Exploratory spatial data analysis (ESDA) was applied through the use of GeoDa™ software version 1.16.0.0 (Spatial Analysis Laboratory, University of Illinois, Urbana Champaign, IL, USA) to determine measures of global spatial autocorrelation, local spatial autocorrelation and to fit spatial regression models (23,24).

**Spatial autocorrelation**: To evaluate the existence of spatial autocorrelation, we used Queen Contiguity matrix that allows for the measurement of nonrandom association between the value of a variable observed in a given geographical units and the value of variables observed in neighboring units (24, 25). Using Global Moran's I index, MPP in each districts were calculated. Moran's I index identifies if the value of MPP tends to be clustered (positive Moran I) or dispersed (negative Moran's I) among geographical areas (24).

Local indicators of spatial association (LISA) clustering method was applied for graphical depiction of spatial autocorrelation. The LISA maps identify significant spatial clusters throughout SNNPRS, with high or low association among the observed values for MPP (25). Clustered areas are categorized according to the pattern of characteristics in adjacent districts. High/high (HH) areas are a set of districts with high value of MPP surrounded by other districts with high value of MPP in univariate analysis. The same sense is applied to low/low (LL) set of districts, where districts with low characteristics are surrounded by other districts with low values for analyzed variables. When the inverse occurs, districts with low value of MPP are surrounded by districts with high value of MPP; LISA maps categorize them as low/high (LH) or high/low (HL) for the opposite pattern.

**Spatial regression**: Spatial autocorrelation occurs when events occurring at different but nearby locations are correlated with each other. This phenomenon is quite likely to be observed for infectious diseases like malaria that is observed within districts. Spatially auto-correlated data should not be analyzed by normal-regression analysis as the correlation violates the basic assumption of ordinary least squares (OLS) regression. Straightforward spatial regression analysis uses a spatial weight matrix and maximum likelihood estimation to minimize the possible bias resulting from spatially auto correlated data (26, 27, 28).

Spatial regression models were introduced to address the spatial dependence existing in the data. The form of the general spatial model is (26)

(1) $$Y=\rho W_{1}Y+X\beta +\mu$$

(2) $$\mu =\lambda W_{2}\mu +\varepsilon \text{ where }\varepsilon \sim MVN(0,\sigma^{2}I_{n})$$

Where Y, the dependent variable, (*n* × 1) is a vector of MPP in a given district, X is a (*n* × *n*) matrix of K explanatory variables, *ρ* and *λ* are spatial autoregressiove coefficients, *W*_1_ and *W*_2_ are (*n* × *n*) spatial weight matrices, *μ* is unobserved error term that incorporates spatial correlation through its first term, *ε* is a (*n* × 1) vector of unobserved error term that are independently and identically distributed, MVN denotes the multivariate normal distribution, and *I_n_* is the (*n* × *n*) identity matrix. In Eq. (1), the model becomes a spatial lag model (SLM) when W2 is 0 and a spatial error model (SEM) when W1 is 0.

In SLM, a dependent variable in a region is subject to a spill-over effect from the dependent variable in the neighboring regions. This effect is accounted for by the spatial with matrix W_1_. On the other hand, in the SEM model, the error in one region is dependent on the error in neighbouring regions through W_2_. In this study, the spatial weight matrices W_1_ and W_2_ were defined using Queen contiguity method of spatial weight matrix propossed by (26) which defines neighbours such that if a portion of boundary (either edge or vertex) between two regions is shared, the corresponding element of spatial weight matrix W_ij_ is 1 and 0 otherwise.

Ethical clearance was obtained from Hawassa University Department of Statistics and permission was taken from Ethiopian Public Health Institute to extract secondary data from records. The data from the case were handled with confidentiality.

## Results

**Figure 1:**
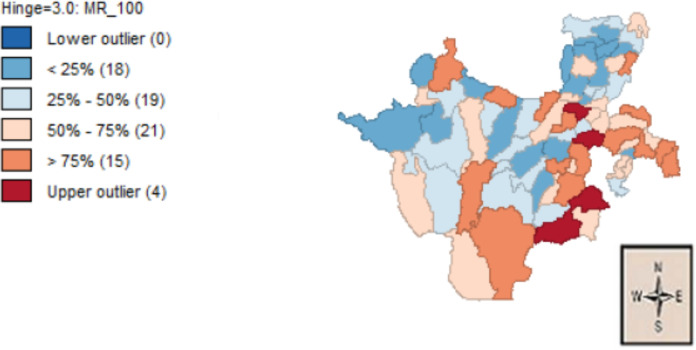
Box plot map for MPP rate in SNNPRS

To identify districts that had below and above average MPP in SNNPRS, a box map was shown in Figure 1. This map shows the location of every district within the overall geographical distribution MPP. The high bright and less bright blue color in the map indicates districts that had MPP lower than average of the region and these districts were clustered around the center, west and north outer border of the region, while the high dark and less dark red color represents districts that had above average MPP which were concentrated in southern, south-east and eastern part of the region.

This box map provided some indication of spatial clustering of MPP in SNNPRS, however the ‘visual inspection’ of maps has long been recognized by cartographers as unreliable in terms of detecting clusters and patterns in the data as human perception is not sufficiently rigorous to assess ‘significant’ clusters and indeed tends to be biased towards finding patterns, even in spatially random data (29). For that purpose, we should turn to a consideration of a global statistics for spatial autocorrelation and the results are presented in the next section.

**Exploratory spatial Data analysis (ESDA) of MPP**: Moran's I statistics was applied to test the presence of global spatial autocorrelation patterns in the distribution of MPP among the districts of the SNNPRS. The value of Moran's I statistics was 0.1147 and significant at 5% level, indicating positive spatial autocorrelation across districts.

This is an indication that the distribution of MPP was not random among the districts rather there is a significant spatial clustering, that is; districts with low MPP (below average) were surrounded by districts with low MPP and/or districts with high MPP (above average) were surrounded by districts with high MPP across the region. This result assumes of globally stationery spatial relationship of MPP in the region; however, it needs a support of high local spatial clusters instead of high local spatial outliers which are indicators of spatial heterogeneity (29).

**Local Measures of Spatial Association (LISA)**: Moran's I statistics, as a global measure of spatial autocorrelation, indicated the presence of significant spatial clustering in the distribution of MPP. Often one wants to move beyond global measures of spatial autocorrelation to identify units that exhibit spatial autocorrelation with their neighbors (30). In this regard, to disaggregate the spatial dependence diagnosed with ESDA and identify local spatial autocorrelation, we employed LISA which were performed via Moran's scatter plot and LISA maps

**Fig 2:**
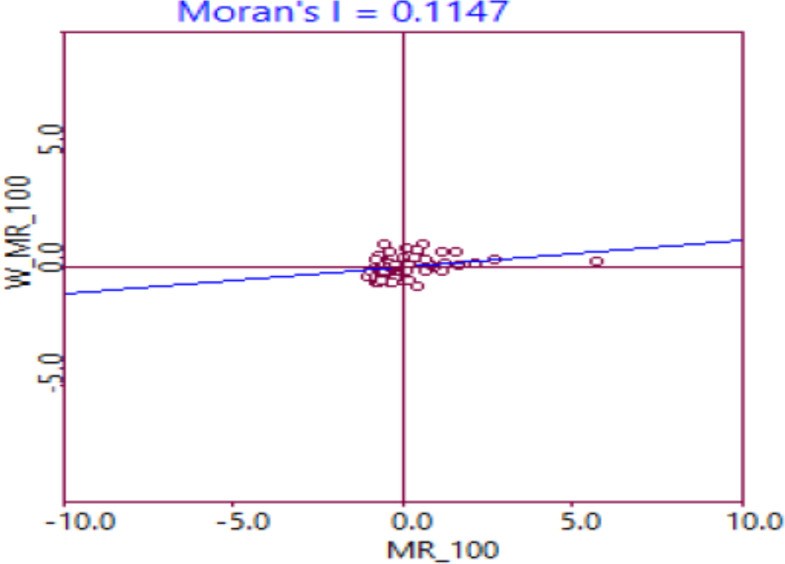
Moran's Scatter Plot

As shown in Figure 2, the Moran scatter plot of MPP, which represent a standardized MPP of a district in the x-axis versus the weighted average (spatial lag) of standardized MPP of its own district in the y-axis, which disaggregate the global spatial autocorrelation into four types of association (HH, HL, LH, LL). Points in quadrant I shows those districts with high MPP (i.e., relative to average of the 76 districts) which were surrounded by districts of high MPP (HH), quadrant II shows districts with low MPP which were surrounded by districts with high MPP (LH), quadrant III shows districts with low MPP which were surrounded by districts with low MPP (LL), and quadrant IV shows districts with high MPP which were surrounded by those districts having low MPP (HL). There are more points in quadrant I and III indicating a positive spatial autocorrelation in the distribution of MPP among districts of the region. However, this needs a formal statistical test to conclude.

**Figure 3:**
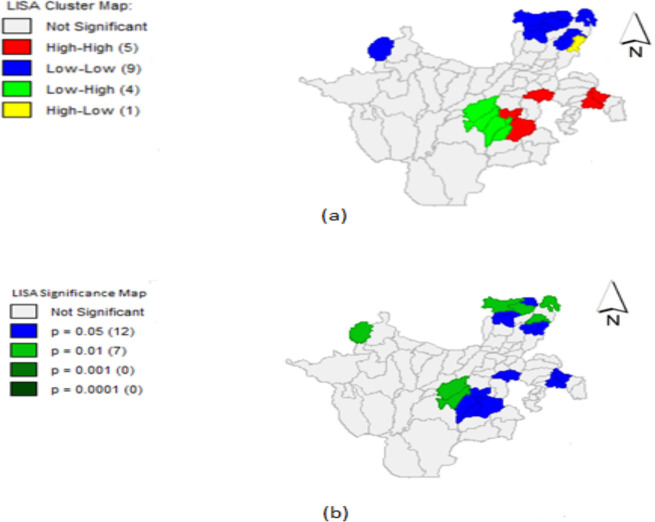
LISA Maps: (a) Cluster Map, (b) Significance Map

Univariate LISA cluster and significance maps of MPP were presented in Figure 3 (a) and Figure 3 (b) respectively.

In LISA cluster map which is useful in identifying the type of spatial association, the HH, LL, LH and HL association of malaria cases were shown in the figure by red, blue, green and yellow colors respectively (Figure 3 (a)). Here when we say high or low values, we are saying high or low values relative to neighboring districts. Positive spatial autocorrelation or clustering of similar values were indicated by high-high or low-low locations where as negative spatial autocorrelation or spatial outlier is indicated by high-low or low-high locations.

LISA significance map (Figure 3 (b)) shows results of local Moran's I test for local spatial autocorrelation patterns of MPP. In the map, the bright blue and green shade corresponds to location of MPP that had significant local spatial autocorrelation at 5% and 1% levels of significance respectively. There were about 19 districts (25%) that had significant local spatial autocorrelation pattern evidencing strong spatial autocorrelation.

Hot spot locations are in the eastern and south-east parts of the region particularly in districts of Deta Daramalo, Arbaminch zuria, Hula, Arbegona and Humbo where as cold spot locations for MPP are around north and northwest parts of the region namely Masha Anderacha, Silti, Dalocha, Enemor Ena Ener, Cheha, Eza Ena Welenie, Gorro, Gumer and Kokir Gedebano districts. Overall, 5 districts had HH, 10 had districts LL, 4 districts had LH and 1 district had HL association of MPP and among districts with significant local spatial association, 25% of them had local spatial clusters. This supports the evidence of positive spatial autocorrelation pattern in distribution of MPP across the region in the results of Moran's I statistics for testing of global spatial autocorrelation.

**Fitting Spatial Regression Models:** Measures of fit and tests that are used to make comparisons between spatial regression models from their respective parts are presented in Table 1. A significant value of likelihood ratio test results together with slightly equal value of log-likelihood test statistic indicates that that both SLM and SEM fit well to the data under consideration.

As recommended by (27), a means of discriminating between spatial lag and spatial error dependence is provided through the use of Lagrange multiplier (LM) diagnostics. The simple versions of LM test are powerful but not robust in local misspecification of the model, so the LM test for spatial lag dependence can be significant even if the form of the spatial dependence resembles spatial error dependence or vice versa. Thus, it is better to look at their robust part so as to come up with the correct identification of the form of spatial dependence in the data.

The results in Table 1 shows that the spatial lag rather than spatial error dependence is evidenced by the robust measures, since the robust Lagrange multiplier test statistic for SLM has relatively higher value than that of SEM counterpart. This indicates that taking SLM as a good fit than the SEM is reasonable.

**Diagnostic tests results of SLM**: The diagnostic tests for the assumptions of spatial autoregressive models are presented in (Table 2). The non-singularity of the design matrix of explanatory variables is diagnosed using condition number. As a rule of thumb, a condition number larger than 30 is considered to be implication for the existence of multicollinearity (31). In Table 2 the condition number is 23.299 which indicate that multicollinearity is not severe problem.

**Table 2:** Spatial Regression Model Assumptions Diagnostics Test of SLM

Test	DF	Value	Probability
**Jarque-Bera test**	2	132	0.0613
**Breusch-Pagan test**	14	33.7866	0.1393
**Koenker-Basset test**	14	9.6257	0.5643
**White test**	119	77.000	0.0361*
**Condition Number**	-	23.299	-

* significant at 5% level

Highly insignificant value 33.78 (p-value 0.1393) of Breusch-Pagan test for hetereschedasticity in the error terms suggests that heteroscedasticity is not serious problem. In addition, from Koenker-Basset and White test, since the p-values for both tests are not significant at the 5% level, we fail to reject the null hypothesis of homoscedasticity and conclude that the variance of the error is constant.

The hypothesis of normality of residuals is also not rejected, since the Jarque-Bera test have p-value of 0.0613 which is greater than 5%. Thus, as diagnostics result from Table 2 supports, the assumption of linearity, no multicollinearity, normality and homoskedasticity were met.

**Maximum likelihood estimation of coefficients of SLM for MPP**: The maximum likelihood estimates of coefficients, their standard errors, test statistic and p-values of factors analyzed in MPP are displayed in Table 3. The effect of individual explanatory variables on the geographical variations of MPP was tested by Z statistics on the control of effects of spatial lag dependence.

**Table 3:** Maximum Likelihood Estimates for Factors of MPP Rate in SLM

Variable	Coefficient	Std. Error	z-value	Probability
***WMR*_100(*ρ*)**	0.3463376	0.1849388	1.872714	0.0412*
**CONSTANT**	-1.489378	3.265984	-2.9161874	0.0359
***DRW*_100**	-0.008026851	0.04664729	-0.1720754	0.86338
**ALTITUDE**	0.003187933	0.003316843	0.9611349	0.33648
**TOI_COV**	-0.1772313	0.09463082	-1.872871	0.06109
***RDTV*_100**	-0.1212584	0.05546446	-2.186236	0.02880*
**NRM**	1.035586	2.670711	3.877568	0.00011*
***FLR*_100**	0.2659137	0.1682745	2.768771	0.00563*
***UMN*_100**	0.0993071	0.03809521	2.606813	0.00914*
***ROF*_100**	0.1006837	0.04977158	2.022915	0.04308*
**NMN**	0.293876	2.039816	2.204984	0.02746*
***ANTMSP*_100**	0.1372591	0.0723726	1.896562	0.0501*
***WAL*_100**	0.1110026	0.2713261	0.4091114	0.68246
**NHHDM**	-1.042806	1.391595	-0.7493599	0.45364
**WEALTHIX**	-2.75393e-005	2.346996e-005	-1.173385	0.24064
***ELEC*_100**	-0.1311861	0.1855111	-0.7071606	0.47947

* significant at 5% level

The estimated coefficient for spatial lag of MPP (*ρ*) was positive and significant indicating that MPP in one district depends directly on the MPP of its neighboring districts. In other words, MPP tend to be more clustered by districts than what would be expected by random distribution. This result supports what we have obtained using Moran's I statistics and cluster map in ESDA of MPP in the previous section. The importance of including spatial lag effects in SLM is supported by the positive and significant value of the coefficient.

Proportion of households living in earth/local dung plastered floor house(FLR_100), proportion of households living under thatched roof house (ROF_100), average number of rooms/person in a given district (NRM), proportion of households who used anti-malaria spray in the last 12 months before the survey (ANTMSP_100), percentage household using mosquito nets (UMN_100) and average number of mosquito nets/person in a given district (NMN) have positive and statistically significant effect on spatial distribution of MPP across districts of SNNPRS. The only variable having negative and statistically significant effect on MPP is the percentage of households living without an access to radio and television (RDTV_100).

Positive effect means that a unit increase in explanatory variable increases MPP in a certain district and its neighboring districts by magnitude of estimate of coefficient for that particular explanatory variable keeping the effect of the other explanatory variables constant, whereas negative effect is to mean that a unit increase in the explanatory variable decreases MPP in a given district and its neighboring districts by a magnitude of estimate of coefficient for that particular explanatory variable keeping the effect of the other explanatory variables constant.

For instance, 1% decreases in percentage of households having no access to radio and television (RDTV_100) in certain district increases MPP in that particular district and its neighboring districts by 0.1213% keeping the effect other variables constant. The parameter estimate of 0.0993 for percentage household using mosquito nets (UMN_100) indicates that 1% decrease in percentage household using mosquito nets decreases the possibility of malaria infection in that particular district and its neighbors by 0.0993% keeping the effect of other explanatory variable constant.

We can also interpret the coefficients of other variables in the same manner as a unit increase/decrease in independent variable increases/decreases MPP in a district (and its neighbor) by magnitude of coefficient estimate controlling for the effect of other explanatory variables.

The parameter estimates for altitude, proportion of households having piped water, proportion of households having protected toilet, proportion of households whose living house wall is mud/stick/wood are not significant in SLM. This means that each of these explanatory variables does not contribute significantly to MPP in SNNPRS. However, the sign of the coefficients suggests the following.

Controlling for the effect of spatial lag and other explanatory variables, altitude is positively related with the value of MPP in districts of SNNPRS indicating that districts with higher value of mean altitude above sea level tend to have higher value of MPP.

## Discussion

This study investigates the spatial pattern of MPP across districts of SNNPRS. A central feature of ESDA is the use of formal statistical tests to assess the degree of spatial randomness observed in the data and spatial autocorrelation test is the most available tool for ESDA of aggregated data. The Moran's I, being the most available measure of global spatial autocorrelation pattern in MPP, showed the existence of positive spatial autocorrelation for MPP. Again we have seen in ESDA of this study that these spatial patterns are not random. The result is consistent with earlier studies that found significant spatial autocorrelations in spatial distribution of malaria prevalence in Ethiopia by (30) and by (32).

The significance of spatially lagged dependent variable (*ρ*) in MPP suggests that neighboring districts prevalence are important determinants of a district's malaria infection possibility. A significant (*ρ*) also indicate that the data under consideration is spatially dependent for morbidity studies and employing OLS models will result in inconsistent estimates due to spatial multiplier bias for the data under consideration. Moreover, these results are an indication that the coefficient estimates and standard errors of the OLS model assuming independent observations may result in spurious results.

The diagnostics results of spatial regression models revealed that both SLM and SEM are equally important in modeling MPP. But based on the less significance of robust Lagrange multiplier (error) residuals in spatial error term, as compared to that of spatial lag model, we found the SLM better than the SEM in fitting spatial regression model for MPP data.

The results of selecting spatial lag model for malaria prevalence as a better fit to the data agrees with what (33, 34) pointed out, i.e fitting classical regression model under presence of spatial dependence may result in inaccurate classical regression model. Also based on measure of fits, SLM was found better than the SEM in predicting the spatial pattern of malaria data, with less Akaike information criterion (AIC) and higher log-likelihood (LIK). This result is consistent with the study done in Cambodia by (35) and in northwestern Peruvian coast by (36). Therefore, spatial lag model was a better way to understand the factors associated with the geographical variations of MPP in SNNPR and we limit the discussion to results of SLM.

Among all the explanatory variables considered in the study, the spatial lags of MPP (WMR_100 (ρ)) have the largest effect on the spatial distribution of MPP relative to other explanatory variables considered under the study. The clustering of the underlying diseases dimensions might be due to a number of reasons including situations that has been applied to groups of areas or socio-economic issues that leading to spatial clustering of MPP.

Proportion of households living in earth/local dung plastered floor house (FLR_100) and proportion of households living under thatched roof house (ROF_100) have significant and positive effect on MPP of districts. This imply that different types of housing materials have an influence on the risk of malaria transmission with those houses constructed of poor quality materials having an increased risk of malaria. This finding is similar with that of in Eastern Rwanda by (37), in northwestern Peruvian coast by (36) and in Ethiopia by (32, 38).

Results also indicated that average number of rooms per person in a given district (NRM), proportion of households who used anti-malaria spray in the last 12 months before EMIS-2011 survey (ANTMSP_100), percentage household using mosquito nets (UMN_100) and average number of mosquito nets per person in a given district (NMN) have positive and statistically significant effect on MPP. The result is consistent with earlier studies in Ethiopia by (32), in northwestern Peruvian by (36) and in Eastern Rwanda by (37). Therefore, using mosquito nets and spraying anti-mosquito treatment on the walls of the house were also found to be a way of reducing the risk of malaria. In addition to this, with the correct use of mosquito nets, anti-mosquito spraying and other preventative measures, like having more rooms in a house, the occurrence of malaria could be decreased.

The coefficient estimate of percentage of households having no access to radio and television is negative and significant at 5% level of significance indicating MPP will be greater in districts having less percentage of households with access to radio and television. This result agrees with the finding of (32) who concluded that households with access to television were at lower risk of malaria compared to households having no television access.

In conclusion, both explanatory spatial data analysis and spatial regression model results revealed positive spatial autocorrelation pattern of MPP meaning that MPP were clustered in space. The eastern and southeast parts of the region are found to be hot spot areas for MPP. Thus, peoples living in these areas are at higher risk of malaria infection.

From results of model specification and measures of fits, the SLM was found to better fit to the data and explain the geographical variations of MPP in the region.

Districts with higher proportion of households living under earth/local dung plastered floor house, higher proportion of households living under thatched roof house and higher proportion of households without an access to radio and television are at higher risk of malaria. Moreover, less number of rooms per person and less mosquito nets per person in households in a given districts puts that particular districts at higher risk of malaria. Spatial risk map also indicated that residents living in the eastern and southeastern parts of the region are at greater risk of malaria infection.

Improving the housing condition of the household is one of the means of reducing the risk of malaria. The implication of the spatial dependence is that, in cases where the decisions on how to allocate funds for interventions needs to have spatial dimension.

The limitation of the study is that the effect of temperature, precipitation and vegetation index is not estimated due to unavailability of the data. Thus further research is recommended by incorporating these variables.

## References

[1] WHO (2019). World malaria report.

[2] Legesse Y, Tegegn Y, Belachew T, Tushune K (2007). Knowledge, Attitude and Practice about Malaria Transmission and Its Preventive Measures among Households in Urban Areas of Assosa Zone, Western Ethiopia. Ethiop J Health Dev.

[3] Deressa W, Olana D, Chiba S (2004). Magnitude of malaria admissions and deaths at hospitals and health centers in Oromia, Ethiopia. Ethiop Med J.

[4] Barofsky J, Chase C, Anekwe T, Farzadfar F (2011). The economic effects of malaria eradication: Evidence from an intervention in Uganda. PGDA Working Paper 7011: Program on the Global Demography of Aging.

[5] Nigatu A, Homa G, Getachew D (2014). Can training health extension workers in the integrated pharmaceutical logistics system (IPLS) be effective, affordable, and opportunistic?. Ethiop Med J.

[6] Gerensea H, Teklay H (2017). Pattern and Trend of Malaria Morbidity and Mortality in Tigray Region, Ethiopia from 2011/12–2014/15. J Bioanal.

[7] Gebre B, Negash Y (2002). Severe malaria among children in Gambella region, western Ethiopia. Ethiop J Health Dev.

[8] Kleinschmidt I, Sharp BL, Clarke GPY, Curtis B, Fraser C (2001). Use of generalized linear mixed models in the spatial analysis of small-area malaria incidence rates in KwaZulu Natal, South Africa. Am J Epidemiol.

[9] Yukich JO, Zerom M, Ghebremeskedl T, Tedios F, Lengeler C (2009). Costs and cost-effectiveness of vector control in Eritrea using treated bed nets. Malaria journal.

[10] Yewhalaw D, Legesse W, Van Bortel W (2009). Malaria and water resource development: the case of Gilgel-Gibe hydroelectric dam in Ethiopia. Malaria Journal.

[11] Tamiru MA, Kassa AW, Beyene BB, Mossie TB, Mekonnen YA (2014). Malaria Outbreak Investigation in Mecha, Dera and Fogera Districts, Amhara Region, Ethiopia. American Journal of Health Research.

[12] Ssempiira J, Nambuusi B, Kissa J (2017). Geostatistical modelling of malaria indicator survey data to assess the effects of interventions on the geographical distribution of malaria prevalence in children less than 5 years in Uganda. PLoS One.

[13] Millar J, Psychas P, Abuaku B (2018). Detecting local risk factors for residual malaria in northern Ghana using Bayesian model averaging. Malar J.

[14] Belay DB, Kifle YG, Goshu AT, Gran JM, Yewhalaw D, Duchateau L, Frigessi A (2017). Joint Bayesian modeling of time to malaria and mosquito abundance in Ethiopia. BMC Infectious Diseases.

[15] Simon C, Moakofhi K, Mosweunyane T (2013). Malaria control in Botswana, 2008–2012: the path towards elimination. Malar J.

[16] Ndiath MM, Cisse B, Ndiaye JL, Gomis JF, Bathiery O, Dia AT (2015). Application of geographically-weighted regression analysis to assess risk factors for malaria hotspots in Keur SOCE health and demographic surveillance site. Malar J.

[17] Ssempiira J, Kissa J, Nambuusi B (2018). The effect of case management and vector-control interventions on space-time patterns of malaria incidence in Uganda. Malar J.

[18] Ssempiira J, Kissa J, Nambuusi B (2018). Interactions between climatic changes and intervention effects on malaria spatio-temporal dynamics in Uganda. Parasite Epidemiology and Control.

[19] Gething PW, Casey DC, Weiss DJ (2016). Mapping Plasmodium falciparum mortality in Africa between 1990 and 2015. New England Journal of Medicine.

[20] Yeshiwondim AK, Gopal S, Hailemariam AT, Dengela DO, Patel HP (2009). Spatial analysis of malaria incidence at the village level in areas with unstable transmission in Ethiopia. International Journal of Health Geographics.

[21] CSA (2012). 2007 Population and Housing Census of Ethiopia: Administrative Report.

[22] EPHI (2011). 2011 Ethiopia National Malaria Indicator Survey.

[23] Anselin L, Syabri I, Kho Y (2006). GeoDa: An Introduction to Spatial Data Analysis. GeoDa Anal.

[24] Anselin L, Longely PA, Goodchild MF, Maguire DJ, Rhind DW (1998). Interactive Techniques and Exploratory Spatial Analysis. Geographical Information Systems: Principles, Techniques, Management and Applications.

[25] Geatan C, Guyon X (2009). Spatial Statistics and Modeling.

[26] Griffith DA (1988). Spatial econometrics: methods and models. Econ Geogr.

[27] LeSage JP, Pace RK (2009). Introduction to Spatial Econometrics.

[28] Rhee KA, Kim JK, Lee YI (2016). Spatial Regression Analysis of Traffic Crashes in Seoul. Accid Anal Prev.

[29] Messner S, Anselin L, Baller R, Hawkins D, Deane G, Tolney S (1999). The Spatial Patterning of County Homicide Rates: an Application of Exploratory Spatial Data Analysis. Journal of Quantitative Criminology.

[30] Goshu AT, Shamenna AT (2016). Spatial Modelling of Fever Prevalence and Suspected Malaria Cases among Children: Cross-sectional Study. International Journal of TROPICAL DISEASE & Health.

[31] Anselin L, Griffith DA (1988). Do Spatial Effects Really Matter in Regression Analysis?. Papers in Regional Science Association.

[32] Ayele DG, Zewotir TT, Mwambi HG (2012). Prevalence and risk factors of malaria in Ethiopia. Malar J.

[33] Anselin L, Griffith DA (1988). Do spatial effects really matter in regression analysis?. Papers in regional sciences.

[34] Griffith DA, Kitchin R, Thrift N (2009). Spatially autoregressive models. International Encyclopedia of Human Geography.

[35] Sluydts V, Heng S, Coosemans M, Van Roey K, Gryseels C, Canier L (2014). Spatial clustering and risk factors of malaria infections in Ratanakiri Province, Cambodia. Malar J.

[36] Aguirre AR, Ponce OJ, Escobar GC (2015). Plasmodium vivax malaria at households: spatial clustering and risk factors in a low endemicity urban area of the northwestern Peruvian coast. Malaria J.

[37] Rulisa S, Kateera F, Bizimana JP (2013). Malaria Prevalence, Spatial Clustering and Risk Factors in a Low Endemic Area of Eastern Rwanda: A Cross Sectional Study. PLoS One.

[38] Molla E, Ayele B (2015). Prevalence of Malaria and Associated Factors in Dilla Town and the Surrounding Rural Areas, Gedeo Zone, Southern Ethiopia. J Bacteriol Parasitol.

